# Non-high-density lipoprotein cholesterol predicts nonfatal recurrent myocardial infarction in patients with ST segment elevation myocardial infarction

**DOI:** 10.1186/s12944-017-0418-5

**Published:** 2017-01-23

**Authors:** Ming Gao, Yang Zheng, Weihua Zhang, Yi Cheng, Lin Wang, Ling Qin

**Affiliations:** 1 The Cardiovascular Center, First Hospital of Jilin University, 71 Xinmin Street, Changchun, 130021 China; 2 Laboratory for Cardiovascular Diseases, Institute of Translational Medicine, Changchun, China; 3 Key Laboratory for Cardiovascular Mechanism of Traditional Chinese Medicine, First Hospital of Jilin University, Changchun, 130021 China

**Keywords:** Recurrent myocardial infarction, ST segment elevation myocardial infarction, Serum transaminase, Primary percutaneous coronary intervention, Lipid

## Abstract

**Background:**

Lipids, which are associated with atherogenesis, clotting, and the fibrinolytic pathway, may be important prognostic indicators of recurrent myocardial infarction. The aim of this study was to determine the predictive value of baseline lipid fractions for nonfatal recurrent myocardial infarction in patients with ST segment elevation myocardial infarction 2 years after primary percutaneous coronary intervention in China.

**Methods:**

Cox proportional-hazards models were used to evaluate the association between potential risk factors, including lipid fractions, and the occurrence of nonfatal recurrent myocardial infarction in 2402 consecutive patients who underwent primary percutaneous coronary intervention for ST segment elevation myocardial infarction.

**Results:**

The cumulative incidence of recurrent myocardial infarction was 2.7% at 1 year, 3.8% at 2 years, and 5.8% at 3 years after percutaneous coronary intervention. The effects of collinearity of lipids were investigated. In concerning the principal components analysis, composing factor 1 (scoring factors were 0.689 for non-HDL, 0.702 for LDL, 0.182 for HDL) which had eigenvalues of 1.86 and explained 62% of the variability among lipid cholesterols was significantly associated with recurrent MI in the final adjusted analysis of the lipid cholesterols principal components. Non-high-density lipoprotein cholesterol was the strongest independent predictor of nonfatal recurrent myocardial infarction. The adjusted hazards ratios for nonfatal recurrent myocardial infarction were 1.26 (95% confidence interval (CI): 1.05–1.51) for non-high-density lipoprotein cholesterol, 1.17 (95% CI: 0.99–1.39) for low-density lipoprotein and 1.15 (95% CI: 0.95–1.40) for HDL. After adjusting for gender and age, the odds ratio for patients in the highest non-high-density lipoprotein cholesterol quartile was 2.10 (95% CI: 1.19–3.72).

**Conclusions:**

Non-high-density lipoprotein cholesterol value is a stronger predictor of nonfatal recurrent myocardial infarction than other lipid risk factors in patients with ST segment elevation myocardial infarction. Moreover, the occurrence of reinfarction after percutaneous coronary intervention was highest for patients in the highest non-high-density lipoprotein cholesterol quartile.

**Trial registration:**

http://www.chictr.org.cn/edit.aspx?pid=13583&htm=4, registration number: ChiCTR-EPC-16008199, date of registration:2013.01.01.

**Electronic supplementary material:**

The online version of this article (doi:10.1186/s12944-017-0418-5) contains supplementary material, which is available to authorized users.

## Background

Primary percutaneous coronary intervention (PCI), the current standard treatment for ST segment elevation myocardial infarction (STEMI), reduces the risk of recurrent myocardial infarction (MI). However, MI reoccurs in 2–6% patients after successful PCI and is associated with poor clinical outcomes [1–3]. Survivors of acute coronary syndromes have a high risk of recurrent events [4], the reasons for which are complex and multifaceted. For instance, stent thrombosis can lead to recurrent MI, although newer durable polymer-based drug-eluting stents have antithrombogenic properties, resulting in a lesser degree of thrombus adhesion [5–7]. Other events that can lead to recurrent MI are the progression of atherosclerosis and lipid-rich plaque rupture [4, 8, 9].

Lipids, which are associated with atherogenesis, clotting, and the fibrinolytic pathway, are important prognostic indicators of recurrent MI [8, 10]. Low-density lipoprotein (LDL) cholesterol is a key risk factor for cardiovascular disease, and current guidelines indicate that first-line therapy should focus on lowering LDL [11–13]. However, prospective cohort studies show that non-high-density lipoprotein (non-HDL) cholesterol is an independent risk factor for mortality among individuals predominately free of coronary heart disease [14, 15], and recently revised National Cholesterol Education Program guidelines recommend that non-HDL and LDL be equally targeted in patients with coronary heart disease [16]. Non-HDL, which is calculated by subtracting high-density lipoprotein (HDL) cholesterol from total cholesterol (TC) [13], includes triglyceride (TG)-rich lipoprotein, LDL, very low-density lipoprotein (VLDL), chylomicron remnants, and intermediate-density lipoprotein (IDL) [17, 18].

Recently, Non-HDL has arose considerable interest and been shown to be an effective predictor of cardiovascular disease, including cardiovascular mortality, in patients with [19–21] and without [15, 22, 23] cardiovascular disease. However, few studies have explored the predictive value of non-HDL after adjusting for collinearity among lipid variables and directly compared varying baseline lipid fractions for predicting nonfatal recurrent MI for coronary artery stenosis among patients with STEMI treated with stents or angioplasty, especially in China. In China, home to one-fifth of the world’s people, there is a rising burden of cardiovascular disease. Therefore, previously studies from Western countries may not be applicable. It is known, racial differences, including genetic factors, life style, and the environmental circumstances will affect long-term outcomes in STEMI patients. Consequently, the aim of this study was to determine whether baseline TG, HDL, non-HDL, or LDL values predict nonfatal recurrent MI in patients with STEMI 2 years after primary PCI in China.

## Methods

### Study population

Data were analyzed from patients with a first STEMI who were admitted to the cardiology department at the First Hospital of Jilin University between January 1, 2013 and December 31, 2014. In accordance with the European Society of Cardiology/American College of Cardiology consensus document [24], we included patients who met at least two of the following criteria: characteristic severe chest pain lasting more than 30 min, electrocardiographic changes, and/or elevation of serum cardiac biomarkers. We excluded patients who had a previous MI, were currently receiving lipid-lowering treatment or drugs, or had insufficient baseline lipid measurement. In total, we included 2402 consecutive STEMI patients who underwent PCI without thrombolysis or conservative therapy. Baseline demographic data, medical history, laboratory data, angiographic results, and clinical variables during hospitalization were retrieved from the department’s electronic database (Table 1). The study protocol was approved by the ethics review board of the First Hospital of Jilin University (No. 2016–263).

**Table 1:** Baseline characteristics of participants

Characteristic	Recurrent MI		*P*-value
	Yes (*n* = 103) *n* (%) or mean ± SD	No (*n* = 2299) *n* (%) or mean ± SD	
Demographic data			
Age (years)	58 ± 12	59 ± 11	0.817
Male	69 (67.0)	1642 (71.4)	0.331
Hospital stay (days)	6.9 ± 2.9	7.2 ± 3.5	0.946
Medical history			
Diabetes mellitus	45 (43.7)	1044 (45.4)	0.152
Hypertension	29 (28.2)	509 (22.1)	0.731
Previous PCI	3 (2.9)	66 (2.9)	0.980
Atrial fibrillation	1 (1.0)	90 (3.9)	0.126
Peripheral vascular disease	3 (2.9)	35 (1.5)	0.261
Arrhythmia (VT/VF)	9 (8.7)	166 (7.2)	0.562
Infarct location by ECG			0.032
Inferior	38 (36.9)	1114 (48.5)	
Anterior	60 (58.3)	1131 (49.2)	
Lateral	5 (4.9)	54 (2.4)	
Killip classification			0.925
I	82 (79.6)	1796 (78.1)	
II	14 (13.6)	312 (13.6)	
III	3 (2.9)	66 (2.9)	
IV	4 (3.9)	125 (5.4)	
Laboratory data			
K^+^ (mmol/L)	3.9 ± 0.5	3.9 ± 0.5	0.181
Na^+^ (mmol/L)	138.6 ± 5.8	139.2 ± 4.1	0.685
Non-HDL (mmol/L)	3.8 ± 1.2	3.5 ± 1.0	0.003
HDL (mmol/L)	1.2 ± 0.3	1.1 ± 0.3	0.053
LDL (mmol/L)	3.1 ± 1.0	2.8 ± 0.8	0.004
TG (mmol/L)	1.8 ± 1.1	1.7 ± 1.2	0.309
Glucose (mmol/L)	7.6 ± 3.2	7.4 ± 3.3	0.368
ALT (unit/L)	46.4 ± 33.1	58.4 ± 114.4	0.171
AST (unit/L)	162.2 ± 162.9	176.4 ± 297.3	0.932
Alkaline phosphatase (unit/L)	71.1 ± 23.1	73.6 ± 28.1	0.309
ɤ-Glutamyl transpeptidase (unit/L)	46.8 ± 43.3	49.1 ± 73.8	0.980
Cardiac troponin I (ng/mL)	23.4 ± 67.7	22.2 ± 62.0	0.646
Creatine kinase MB (ng/ml)	31.7 ± 53.4	40.8 ± 77.7	0.415
NT-proBNP (pg/mL)	1595.7 ± 2259.3	1889.2 ± 4908.0	0.967
Number of stents	1.2 (0.8)	1.2 (0.8)	0.605
Method of reperfusion			0.046
Balloon angioplasty	18 (17.5)	255 (11.1)	
Drug-eluting stent implantation	85 (82.5)	2044 (88.9)	
Thrombus aspiration	9 (8.7)	280 (12.2)	0.294
Temporary pacemaker	3 (2.9)	137 (6.0)	0.197
Late PCI (>12 h after symptom onset)	65 (63.1)	1352 (58.8)	0.386
Discharge medications			
Aspirin	100(97.1)	2263(98.4)	0.290
Platelet P2Y_12_ inhibitor	69(67.0)	1502(65.3)	0.729
Statins	101(98.1)	2276(99.0)	0.357
Beta-blocker	71(68.9)	1477(64.3)	0.331
ACE inhibitor or ARB	68(66.0)	1317(57.3)	0.079
Calcium blockers	6(5.8)	128(5.6)	0.911
Diuretic	34(33.0)	750(32.6)	0.935
All-cause mortality at 2 years	7 (6.8)	119 (5.2)	0.471

*SD* standard deviation, *PCI* percutaneous coronary intervention, *VT/VF* ventricular tachycardia/fibrillation, *ECG* electrocardiogram, *non-HDL* non-high-density lipoprotein, *HDL* high-density lipoprotein, *LDL* low-density lipoprotein, *ALT* alanine aminotransferase, *AST* aspartate aminotransferase, *NT-proBNP* N-terminal pro-brain natriuretic peptide, *ACE* angiotensin-converting enzyme, *ARB* angiotensin receptor blocker

### Primary PCI protocol

Before PCI, patients were administered aspirin (loading dose of 300 mg and maintenance dose of 100 mg/day), clopidogrel (loading dose of 600 mg and maintenance dose of 75 mg/day), and intravenous unfractionated heparin (70 U/kg bolus). Coronary angiography was performed using standard techniques. Thrombus aspiration, a temporary pacemaker, and/or an intra-aortic balloon pump were used at the surgeon’s discretion. Standard management was provided by responsible physicians. Generally, patients received aspirin, atorvastatin/rosuvastatin, clopidogrel, a β-blocker, angiotensin-converting enzyme inhibitors/angiotensin II receptor blockers and diuretics. Dural antiplatelet therapy was prescribed for at least 1 year in all patients with successful drug-eluting stents implantation.

### Biochemical analysis

Baseline TC, TG, HDL, glucose, and LDL values were measured directly in plasma after fasting. All blood samples were obtained at the time of hospital admission and were analyzed in the certified laboratory department of the First Hospital of Jilin University.

### Clinical follow-up

A diagnosis of recurrent MI required two of the following criteria: ischemic symptoms for at least 30 min, electrocardiographic changes and creatine kinase-MB value at least twice the upper limit of normal, or troponin I value at least twice the upper limit of normal. Troponin I levels were not used to diagnose recurrent MI within 10 days of the index MI. Follow-up data were collected from hospital records and telephone interviews after discharge until death or April 1, 2016, whichever occurred first. Mortality data for patients who were lost to telephone follow-up were obtained from computerized records of the population registry bureau.

### Statistical analysis

Continuous variables are presented as mean ± standard deviation (SD), and categorical variables are presented as frequency and percentage. Baseline continuous and categorical variables for patients with and without recurrent MI during follow-up were compared using Wilcoxon’s rank-sum tests and Chi-square tests, respectively. Cox proportional hazard models were used to evaluate associations between baseline lipid levels and clinical characteristics and recurrent MI at 2-year follow-up. Potential confounding factors were entered into the multivariate predictors of recurrent MI if they were clinical relevant or showed univariate differences between groups. Included confounders were lipid, infarct location, diabetes mellitus, atrial fibrillation, peripheral vascular disease, method of reperfusion, thrombus aspiration, temporary pacemaker and number of stents. The lipid variables were correlated in multivariate analysis and potentially resulting in multicollinearity. Principle component analysis was performed in order to investigate the effects of collinearity expected to occur among lipid cholesterol and to determine weights for the included variables. The variables which contributed most to the variance were then selected to be included in the regression analysis. Multivariate models were constructed for lipid risk factor adjusting for other predictors in univariate analyses to calculate multiple-adjusted Hazard ratios (HRs) and their 95% confidence intervals (CIs). According to common practice in principal component analysis, principal composing factors 1 and 2 were retained as covariates in the multivariate models of recurrent MI. Regression coefficients were calculated to estimate the HRs associated with 1-SD higher baseline values of each baseline lipid fraction: 1.20 mmol/L TG, 1.03 mmol/L non-HDL, 0.83 mmol/L LDL, and 0.30 mmol/L HDL. Model 1 was adjusted for age and gender, and model 2 was adjusted for age, gender, Killip classification, prior PCI, diabetes mellitus, hypertension, stroke, atrial fibrillation, thrombus aspiration, arrhythmia (ventricular tachycardia/fibrillation (VT/VF)), and method of reperfusion. Model 3 was with variables in Model 2, as well as with additional lipid fraction (per SD change) of non-collinearity. Lipid values were categorized into quartiles (≤25th, 25th to <50th, 50th to <75th, and ≥75th percentiles). Patients with lipid values in the lowest quartile were used as a reference to assess associations between lipid fractions and recurrent MI, adjusting for covariates. For all analyses, a two-sided *P*-value <0.05 was considered statistically significant. All analyses were conducted using Stata software, version 12 (Stata Corp., College Station, TX).

## Results

### Incidence of nonfatal recurrent MI

**Fig. 1:**
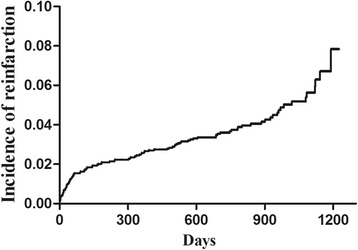
Incidence of recurrent MI during long-term follow-up. The incidence of recurrent MI was 2.7% at 1 year, 3.8% at 2 years, and 5.8% at 3 years

Of the 2402 patients included in this study, there were 129 deaths during the median 2.2 years of follow-up (range: 30–1226 days). A total of 102 patients suffered recurrent nonfatal MI. The incidence of recurrent MI was 2.7% at 1 year, 3.8% at 2 years, and 5.8% at 3 years (Fig. 1).

### Risk factors for recurrent MI

Baseline data from patients with and without recurrent MI, including demographic characteristics, medical history, laboratory data, culprit vessel, and PCI procedure, are shown in Table 1. In concerning the principal components analysis, composing factor 1 had eigenvalues of 1.86 and explained 62% of the variability among lipid cholesterols and composing factor 2 had eigenvalues of 0.98 and explained 33%. Table 2 presented the scoring factors for each variable composing factor 1 and 2. Composing factor 1 was significantly associated with recurrent MI in the final adjusted analysis of the lipid cholesterols principal components (Table 3). Compared with patients without recurrent MI, patients with recurrent MI had significantly higher non-HDL and LDL values.

**Table 2:** Scoring factors for principle components

Variable	Scoring factor	
	Composing Factor 1	Composing Factor 2
Non-HDL	0.689	−0.204
LDL	0.702	−0.053
HDL	0.182	0.978

*non-HDL* non-high-density lipoprotein, *LDL* low-density lipoprotein

**Table 3:** Predictors of recurrent MI

Category	Multivariate Analysis		
	Adjusted HR	95% CI	*P*-value
Composing Factor 1	1.19	1.05–1.35	0.008
Composing Factor 2	1.13	0.94–1.36	0.202
Age	1.00	0.98–1.01	0.585
Gender	0.81	0.52–1.26	0.356
Infarct location by ECG	1.48	1.04–2.11	0.031
Diabetes mellitus	1.45	0.93–2.24	0.100
Atrial fibrillation	0.28	0.04–2.01	0.204
Peripheral vascular disease	1.94	0.60–6.21	0.266
Method of reperfusion	0.45	0.23–0.88	0.020
Thrombus aspiration	0.81	0.40–1.64	0.559
Temporary pacemaker	0.70	0.21–2.31	0.554
Number of stents	1.15	0.86–1.55	0.344

*HR* hazard ratio, *CI* confidential interval, *non-HDL* non-high-density lipoprotein, *LDL* low-density lipoprotein. *ECG* electrocardiogram

Patients with anterior MI, as detected by electrocardiogram (ECG), had a significantly higher rate of MI recurrence than those with inferior or lateral MI. The method of reperfusion also differed significantly between patients with and without recurrent MI. Reinfarction rates did not differ by gender or age. No associations were observed between recurrent MI and diabetes mellitus, hypertension, previous PCI, atrial fibrillation, peripheral vascular disease, arrhythmia (VT/VF), Killip classification, number of stents, thrombus aspiration, late PCI (>12 h after symptom onset), a temporary pacemaker or discharge medications. Multivariate analysis showed that lipid cholesterols, infarct location by ECG, and successful drug-eluting stent implantation were significant independent baseline predictors of nonfatal recurrent MI at 2-year follow-up (Table 3).

### Lipid stratification and recurrent MI

Multivariate regression models examining baseline lipid risk factors showed that nonfatal recurrent MI was most strongly associated with non-HDL followed by LDL (Table 4) after adjusting for age and gender (model 1) or diabetes mellitus, hypertension, infarct location, method of reperfusion, previous PCI, atrial fibrillation, arrhythmia (ventricular tachycardia/fibrillation), Killip classification, and thrombus aspiration (model 2) and variable in model 2 as well as with additional lipid fractions (per SD change) of non-collinearity (model 3).

**Table 4:** Comparison of lipoprotein cholesterol levels in predicting non-fatal recurrent MI

Variable	Model 1 HR (95% CI)	*P*-value	Model 2 HR (95% CI)	*P*-value	Model 3 HR (95% CI)	*P*-value
Non-HDL	1.27 (1.08–1.50)	0.032	1.26 (1.06–1.49)	0.005	1.26 (1.05–1.51)	0.006
LDL	1.22 (1.03–1.44)	0.087	1.20 (1.02–1.42)	0.010	1.17 (0.99–1.39)	0.013
HDL	1.17 (1.00–1.40)	0.175	1.18 (0.99–1.40)	0.016	1.15 (0.95–1.40)	0.006

*HR* hazards radio, *CI* confidence interval, *non-HDL* non-high-density lipoprotein cholesterol, *HDL* high-density lipoprotein cholesterol, *LDL* low-density lipoprotein cholesterol, *TG* triglyceride. Adjusted HRs with 95% CIs of lipid fraction per SD change (1.20 mmol/L TG, 1.03 mmol/L non-HDL, 0.83 mmol/L LDL, 0.30 mmol/L HDL) interval for different models

In the Adult Treatment Panel III report of the National Cholesterol Education Program [13], non-HDL as a secondary target should be limited to patients with elevated serum TG values (>200 mg/dl). When our analysis was restricted to patients with TG values ≤200 mg/dl, the adjusted ORs were 1.49 (95% CI: 0.83–2.70) for non-HDL and 0.88 (95% CI: 0.45–1.72) for LDL. Thus, non-HDL was a stronger predictor of nonfatal recurrent MI than other lipid risk factors in all models.

**Fig. 2:**
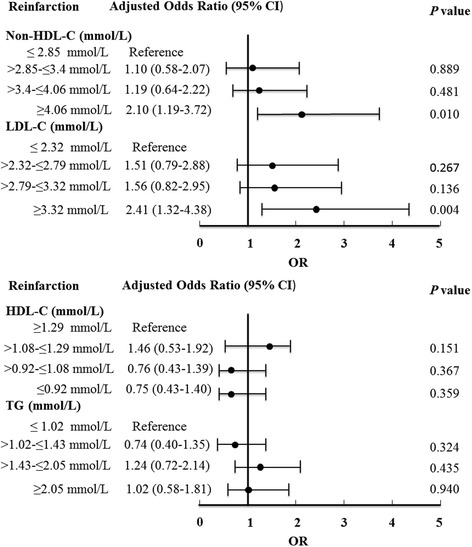
Association between baseline lipid values and incidence of nonfatal recurrent MI. non-HDL, non-high-density lipoprotein; LDL, low-density lipoprotein; HDL, high-density lipoprotein; TG, triglyceride; CI, confidence interval; OR, odds radio

We further categorized lipid values into quartiles (Fig. 2, Additional file 1: Table S1). Patients in the highest non-HDL quartile had the highest OR for recurrent MI in both univariate and multivariate models. Similar results were observed for LDL quartiles, whereas no associations were found between HDL and TG values and risk of recurrent MI. Furthermore, there was a linear increase in the log OR for recurrent MI with increasing non-HDL and LDL quartiles, suggesting a linear relationship between non-HDL and LDL values and recurrent MI.

## Discussion

Within this cohort analysis of STEMI patients with baseline lipid measurements, lipid cholesterols were an independent predictor of nonfatal recurrent MI during long-term follow-up after PCI in China, even when controlling for several demographic and clinical variables known to influence patient outcome. The occurrence of reinfarction was highest for patients with STEMI undergoing primary PCI in the highest non-HDL cholesterol quartile and non-HDL independently predicted nonfatal recurrent MI in a dose-dependent manner.

### Incidence of recurrent MI and prognostic implication

The 1-year MI recurrence rate for our patients was lower than that previously reported for patients with acute MI treated with primary PCI (6%) [25]. However, in a meta-analysis of 23 trials, including patients with STEMI who underwent primary angioplasty, the 1-year MI recurrence rate was 3%, which is closer to our findings [26]. Our results also support an increasing risk of recurrent MI over time, with an incidence 2.15 times higher at 3 years than at 1 year after the index MI. Although MI recurrence rates vary among studies employing different definitions, sample sizes, and follow-up periods, our results are consistent with those of previous studies indicating that the risk of recurrent MI is not eliminated after PCI despite rapid advances in percutaneous therapy over the past decades due to improvements in stent platforms and implantation techniques, reductions in stent thrombosis, and the use of adjunctive medical therapy [27–30]. However, prior studies had demonstrated the superiority of stent implantation over balloon angioplasty alone in the treatment of acute MI [31–33]. Especially, drug-eluting stents further decreased repeat revascularization of target lesion or stent thrombosis and target-vascular infarction than bare-metal stents in patients with STEMI [28, 30]. Our results were consistent with those of others. We found that successful drug-eluting stent implantation reduced the risk of recurrent MI compared with balloon angioplasty alone, as patients with balloon angioplasty alone usually had complicated or irregular coronary lumen or diffuse atherosclerotic lesions [33].

### Baseline lipid values and nonfatal recurrent MI

We found that non-HDL value was an independent predictor of nonfatal recurrent MI in patients with STEMI after PCI. In comparing with other lipid fractions, non-HDL value reflects the TG-rich lipoprotein content of several proatherogenic subfractions, including VLDL, IDL, and chylomicron remnants. Several studies report that IDL and small VLDL values, but not LDL value, correlates with the progression of atherosclerosis [34, 35]. In the National Heart, Lung, and Blood Institute Type II Coronary Intervention Study, TG-rich lipoproteins were associated with a faster progression of atherosclerotic lesions [36]. This previous study reports that the change in IDL at 2 years predicted cardiovascular disease progression at 5 years; this association remained significant after adjusting for treatment assignment. Although the molecular mechanisms of this association are not fully understood, they are becoming increasingly clear. Small TG-rich lipoproteins can be directly taken up by macrophages to produce foam cells and are intimately associated with clotting and the fibrinolytic pathway, thereby contributing to lesion progression, plaque rupture, and clinical coronary events [8, 37]. Our results confirmed that the risk of recurrent MI was highest for patients in the highest non-HDL quartile.

By contrast, non-HDL was not a significant independent risk factor for subsequent all-cause mortality. This might be explained in part because patients with lipoprotein measurements undergoing primary angiography are less sick than those with surgical contraindications or who died before lipid measurements could be obtained. In addition, the nutraceuticals and functional substances contained in food had already been considered to influence lipids levels beyond common clinical treatment [38]. Moreover, the influences of genetics and environmental factors on the lipid levels of individuals, the dosage of statin or other prescription drug, adherence to therapy and cardiac rehabilitation participation have important roles on the overall cardiovascular risk [39–41]. Our results are in accord with those of a previous study that did not detect a relationship between non-HDL and cardiovascular disease outcomes in a cohort of patients with prevalent coronary artery disease [42]. However, a recent systematic review of studies, including patients with multiple risk factors for cardiovascular disease, found no evidence that non-HDL markers predict the occurrence of cardiovascular events [43]. Therefore, there is a need for more research in this field.

Some limitations of this study should be taken into consideration. First, this is a retrospective analysis of a consecutive cohort of patients treated with primary angioplasty from a single center which located in Northeast China and the data may not reflect the general population of STEMI patients. Other P2Y12 were not available except for clopidogrel during the study period. However, patient data were imputed electronically by a relatively constant group of attending physicians; the overall strategic management of patients, including PCI techniques and devices used during the procedure, was more homogeneous than would be in a multi-centered study. Second, sudden death that may have been caused by fatal recurrent MI, and cases of non-symptomatic recurrent MI, may have been overlooked, meaning that the actual incidence of nonfatal recurrent MI was probably higher than that observed in our study. However, these limitations are balanced by our continuous admission and avoidance of ascertainment bias that occurs in clinical studies using selected patients. Third, although we used multivariate models to adjust for covariates, it is possible that unmeasured confounding variables may have affected the relationship between lipid values and the incidence of nonfatal recurrent MI.

## Conclusions

To our knowledge, this is the first study to investigate the relationship between baseline lipid values and nonfatal recurrent MI in patients with STEMI who were treated with primary PCI. Of all lipid fractions, non-HDL was the strongest predictor of recurrent MI. Moreover, the occurrence of reinfarction after PCI was highest for patients in the highest non-HDL quartile.

## Additional file

Additional file 1: Table S1. Associations between baseline lipid values with recurrent MI. (DOC 74 kb)

## Abbreviations

CK: Creatine kinase
ECG: Electrocardiogram
HDL: High-density lipoprotein
IDL: Intermediate density lipoprotein
LDL: Low-density lipoprotein
MI: Myocardial infarction
non-HDL: Non-high-density lipoprotein
PCI: Percutaneous coronary intervention
STEMI: ST segment elevation myocardial infarction
TC: Total cholesterol
TG: Triglyceride
VLDL: Very low-density lipoprotein
VT/VF: Ventricular tachycardia/fibrillation
