# Supplementary material for: Non-high-density lipoprotein cholesterol predicts nonfatal recurrent myocardial infarction in patients with ST segment elevation myocardial infarction
Source: Lipids Health Dis. 2017 Jan 23;16:20. doi: 10.1186/s12944-017-0418-5 (PMC5260128; doi:10.1186/s12944-017-0418-5)
Supplement: Additional file 1: Table S1. — Associations between baseline lipid values with recurrent MI. (DOC 74 kb) [file 12944_2017_418_MOESM1_ESM.doc]

**Additional file 1: Table S1.** **Associations between baseline lipid values with recurrent MI.**

|  | | *Association between non-HDL and recurrent MI* | | | | | | |  |  |  |
| --- | --- | --- | --- | --- | --- | --- | --- | --- | --- | --- | --- |
| **Category** | **Non-HDL** | | **Crude univariate model** | | | **Adjusted for age and sex** | | | **Adjusted for all covariates** | | |
|  |  |  | **OR** | **95% CI** | **P-value** | **OR** | **95% CI** | **P-value** | **OR** | **95% CI** | **P-value** |
| Non-HDL (1)^a^ | ≤2.85 | | *1 (ref)* |  | 0.013 | *1 (ref)* |  | 0.045 | *1 (ref)* |  | 0.011 |
| Non-HDL (2) | >2.85 to ≤3.4 | | 1.05 | 0.56–2.00 | 0.871 | 1.10 | 0.58–2.07 | 0.889 | 0.98 | 0.51–1.86 | 0.947 |
| Non-HDL (3) | >3.4 to ≤4.06 | | 1.27 | 0.69–2.35 | 0.438 | 1.19 | 0.64–2.22 | 0.481 | 1.21 | 0.65–2.25 | 0.550 |
| Non-HDL (4) | ≥4.06 | | 2.19 | 1.25–3.83 | 0.006 | 2.10 | 1.19–3.72 | 0.010 | 1.20 | 1.13–3.54 | 0.017 |
|  | | *Association between LDL and recurrent MI* | | | | | | |  |  |  |
| **Category** | **LDL** | | **Crude univariate model** | | | **Adjusted for age and sex** | | | **Adjusted for all covariates** | | |
|  |  |  | **OR** | **95% CI** | **P-value** | **OR** | **95% CI** | **P-value** | **OR** | **95% CI** | **P-value** |
| LDL (1)^a^ | ≤2.32 | | *1 (ref)* |  | 0.018 | *1 (ref)* |  | 0.048 | *1 (ref)* |  | 0.012 |
| LDL (2) | >2.32 to ≤2.79 | | 1.45 | 0.76–2.79 | 0.257 | 1.51 | 0.79–2.88 | 0.267 | 1.42 | 0.74–2.74 | 0.292 |
| LDL (3) | >2.79 to ≤3.32 | | 1.65 | 0.88–3.12 | 0.119 | 1.56 | 0.82–2.95 | 0.136 | 1.61 | 0.85–3.05 | 0.148 |
| LDL (4) | ≥3.32 | | 2.47 | 1.36–4.49 | 0.003 | 2.41 | 1.32–4.38 | 0.004 | 2.32 | 1.27–4.24 | 0.006 |
| *Association between HDL and recurrent MI* | | | | | | | | | | | |
| **Category** | **HDL** | | **Crude univariate model** | | | **Adjusted for age and sex** | | | **Adjusted for all covariates** | | |
|  |  |  | **OR** | **95% CI** | **P-value** | **OR** | **95% CI** | **P-value** | **OR** | **95% CI** | **P-value** |
| HDL (4)^a^ | ≥1.29 | | *1 (ref)* |  | 0.059 | *1 (ref)* |  | 0.095 | *1 (ref)* |  | 0.218 |
| HDL (3) | >1.08 to ≤1.29 | | 1.45 | 0.86–2.43 | 0.156 | 1.46 | 0.53–1.92 | 0.151 | 1.48 | 0.88–2.49 | 0.143 |
| HDL (2) | >0.92 to ≤1.08 | | 0.76 | 0.42–1.38 | 0.368 | 0.76 | 1.23–3.81 | 0.367 | 0.77 | 0.42–1.42 | 0.410 |
| HDL (1) | ≤0.92 | | 0.76 | 0.42–1.38 | 0.368 | 0.75 | 0.76–2.58 | 0.359 | 0.77 | 0.42–1.43 | 0.413 |
| *Association between TG and recurrent MI* | | | | | | | | | | | |
| **Category** | **TG** | | **Crude univariate model** | | | **Adjusted for age and sex** | | | **Adjusted for all covariates** | | |
|  |  |  | **OR** | **95% CI** | **P-value** | **OR** | **95% CI** | **P-value** | **OR** | **95% CI** | **P-value** |
| TG (1)^a^ | ≤1.02 | | *1 (ref)* |  | 0.309 | *1 (ref)* |  | 0.417 | *1 (ref)* |  | 0.559 |
| TG (2) | >1.02 to ≤1.43 | | 0.75 | 0.41–1.38 | 0.358 | 0.74 | 0.40–1.35 | 0.324 | 0.69 | 0.37–1.28 | 0.237 |
| TG (3) | >1.43 to ≤2.05 | | 1.29 | 0.76–2.22 | 0.343 | 1.24 | 0.72–2.14 | 0.435 | 1.14 | 0.66–1.98 | 0.632 |
| TG (4) | ≥2.05 | | 1.08 | 0.62–1.89 | 0.777 | 1.02 | 0.58–1.81 | 0.940 | 0.96 | 0.53–1.72 | 0.882 |

OR, odds ratio; CI, confidence interval; ^a^Quartiles: (1) <25th percentile, (2) 25th to <50th percentile, (3) 50th to <75th percentile, (4) ≥75th percentile; non-HDL, non-high-density lipoprotein; HDL, high-density lipoprotein; LDL, low-density lipoprotein; TG, triglyceride.
